# Investigation of the Interaction between the Large and Small Subunits of Potato ADP-Glucose Pyrophosphorylase

**DOI:** 10.1371/journal.pcbi.1000546

**Published:** 2009-10-30

**Authors:** Ibrahim Barıs, Aytug Tuncel, Natali Ozber, Ozlem Keskin, Ibrahim Halil Kavakli

**Affiliations:** Department of Chemical and Biological Engineering, College of Engineering, Koc University, Rumeli Feneri Yolu, Istanbul, Turkey; National Cancer Institute, United States of America and Tel Aviv University, Israel

## Abstract

ADP-glucose pyrophosphorylase (AGPase), a key allosteric enzyme involved in higher plant starch biosynthesis, is composed of pairs of large (LS) and small subunits (SS). Current evidence indicates that the two subunit types play distinct roles in enzyme function. Recently the heterotetrameric structure of potato AGPase has been modeled. In the current study, we have applied the molecular mechanics generalized born surface area (MM-GBSA) method and identified critical amino acids of the potato AGPase LS and SS subunits that interact with each other during the native heterotetrameric structure formation. We have further shown the role of the LS amino acids in subunit-subunit interaction by yeast two-hybrid, bacterial complementation assay and native gel. Comparison of the computational results with the experiments has indicated that the backbone energy contribution (rather than the side chain energies) of the interface residues is more important in identifying critical residues. We have found that lateral interaction of the LS-SS is much stronger than the longitudinal one, and it is mainly mediated by hydrophobic interactions. This study will not only enhance our understanding of the interaction between the SS and the LS of AGPase, but will also enable us to engineer proteins to obtain better assembled variants of AGPase which can be used for the improvement of plant yield.

## Author Summary

ADP-glucose pyrophosphorylase (AGPase) is a key heterotetrameric allosteric enzyme involved in plant starch biosynthesis. In this study, we have applied computational and experimental methods to identify critical amino acids of the AGPase large and small subunits that interact with each other during the heterotetrameric structure formation. During the comparison of the computational with the experimental results we also noted that the backbone energy contribution of the interface residues is more important in identifying critical residues. This study will enable us to use a rational approach to obtain better assembled mutant AGPase variants and use them for the improvement of the plant yield.

## Introduction

ADP-glucose pyrophosphorylase (AGPase) is a key regulatory allosteric enzyme involved in starch biosynthesis in higher plants. It catalyzes the rate limiting reversible reaction and controls the carbon-flux in the α-glucan pathway by converting Glucose-1-phosphate and ATP to ADP-glucose and pyrophosphate using Mg^2+^ as the cofactor [1]–[3]. Regulation of almost all AGPases depends on the 3-phosphoglyceric acid to inorganic phosphate ratio (3PGA/Pi ). While 3-PGA functions as the main stimulator, Pi inhibits the activity of enzyme [3]–[5]. Plant AGPases consist of pairs of small (SS, or α) and large (LS, or β) subunits thereby constituting a heterotetrameric structure (α_2_β_2_). These two subunits are encoded by two distinct genes [6]. In potato tuber AGPase the sequence identity between the different subunits is 53% suggesting a common ancestral gene [7],[8]. The molecular weights of tetrameric AGPases range from 200 to 240 kDa depending on the tissue and plant species. Specifically, molecular weights of LS and SS in potato tuber AGPase are 51 kDa and 50 kDa, respectively [6]. It was found that SS and LS have different roles in the enzyme functionality. SS was shown to have both catalytic and regulatory functions whereas LS is mainly responsible for regulating the allosteric properties of SS [9]–[12]. These results were also supported by the studies that showed LS was incapable of assembling into a catalytically active oligomeric structure, whereas SS was able to form a homotetramer with catalytic properties [9],[13]. However, this SS homotetramer showed defective properties in terms of catalysis and regulation. It required higher concentrations of 3-PGA for activation and was more sensitive to Pi inhibition. These results suggested that LS was essential for the enzyme to function efficiently [11],[14],[15]. Alternatively, recent studies have indicated that the LS may bind to substrates glucose-1 phosphate and ATP. The binding of the LS to substrates may allow the LS to interact cooperatively with the catalytic SS in binding substrates and effectors and, in turn, influence net catalysis [12], [16]–[18]. In addition, specific regions from both the LS and the SS were found to be important for subunit association and enzyme stability [15]. Also, using chimeric maize/potato small subunits, Cross et al. [19] found a polymorphic motif in the SS which is critical for subunit interaction. They have concluded that a 55-amino acid region between the residues 322–376 directly interacts with LS and significantly contributes to the overall enzyme stability.

Recently crystal structure of SS was found in a homotetrameric form by Jin et al. [20]. Neither the LS nor the heterotetrameric AGPase (α_2_β_2_) structure have been solved yet. This is due to the difficulty of obtaining AGPase in stable form. However, it is critical to elucidate the native heterotetrameric AGPase structure and identify the key residues taking place in subunit-subunit interactions to obtain a more detailed picture of the enzyme. Understanding the structure and the hot spot residues in the subunit interface will enable us to manipulate the native enzyme to get a stable form which can be utilized for improving the yield of crops. The feasibility of such an approach has been shown previously [21],[22] . We modeled the LS structure of potato tuber AGPase and proposed a model for the heterotetrameric AGPase [23]. In this study, we extended our previous work by examining our AGPase model to identify important residues mediating the interactions between the LS and the SS both by computational and experimental techniques. Based on Molecular mechanics generalized born surface area (MM-GBSA) method, two distinct LS domains are involved in LS-SS subunit interaction. The residues of the potato AGPase LS Asn^97^, Pro^327^, Ile^330^, Ile^335^, Ile^339^, Ile^340^, and His^342^ are involved in lateral interaction with the potato AGPase SS whereas residues Arg^45^, Arg^88^, Arg^92^, and Trp^135^ are involved in longitudinal interaction with the potato AGPase SS. The effect of these mutations on the interactions of the LS and the SS of potato AGPase were further characterized *in vivo* using the bacterial complementation and the yeast two-hybrid methods. Also, experimental results indicated that the backbone **ΔG_binding_** energy of the interface amino acids is a decisive parameter for the subunit-subunit interaction rather than side chain **ΔG_binding_** or total **ΔG_binding_** energies. This study will highlight the important structural aspects of AGPase structure and provide insights for further attempts to engineer a more functional form of the enzyme.

## Results

### Free Energy Decomposition of Lateral (D1) and Longitudinal (D2) Dimers

**Figure 1:**
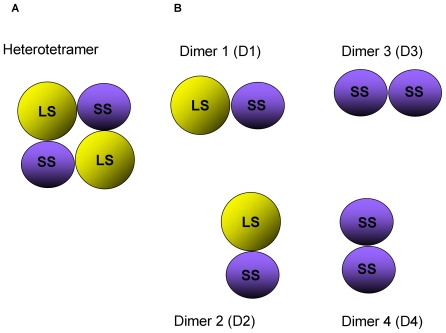
Schematic representation of heterotetrameric AGPase structure. (A) Simplified two dimensional version of the heterotetrameric model of potato AGPase native structure. (B) Dimers heterotetrameric structure SS-LS.

To determine the critical amino acid residues of the potato AGPase LS that interact with potato AGPase SS, we performed MM-GBSA method which calculates the binding free energy and decomposes the energy at the amino acid level. The binding free energy differences for the longitudinal (D2) and lateral (D1) dimers of the modeled heterotetramer [23] (see **Figure 1**) obtained from MM-GBSA method are shown in **Table 1**. It is observed that in all of the dimeric interactions, favorable ΔE_elec_ terms are compensated by unfavorable ΔG_polar_ terms. Hence, total electrostatic interactions ΔG_elec_, favor binding of subunits. Contributions from van der Waals and non-polar solvation energies also favor interactions thus being the major forces that drive the association of subunits. These results are in agreement with our previous work [23].

**Table 1:** Binding free energy components (kcal/mol) for each of the dimers averaged over the 200 snapshots.

	Dimer 1	Dimer 2	Dimer 3	Dimer 4
**ΔE_elec_**	−508.73 (2.88)	−348.80 (3.06)	−135.23 (2.32)	−391.30 (2.79)
**ΔE_VDW_**	−187.61 (0.54)	−103.68 (0.59)	−174.80 (0.63)	−101.01 (0.58)
**ΔE_int_**	0.01 (0.01)	0.01 (0.01)	0.01 (0.01)	0.01 (0.01)
**ΔG_gas_**	−696.33 (3.04)	−452.47 (3.17)	−310.02 (2.58)	−492.30 (2.66)
**ΔG_non-polar_**	−18.48 (0.05)	−11.52 (0.04)	−17.25 (0.04)	−11.83 (0.03)
**ΔG_polar_**	581.13 (2.81)	417.69 (2.75)	209.74 (2.15)	456.11 (2.56)
**ΔG_sol_**	562.65 (2.78)	406.17 (2.74)	192.50 (2.13)	444.28 (2.56)
**ΔG_elec_**	72.40 (0.54)	68.88 (0.66)	74.52 (0.56)	64.81 (0.64)
**ΔG_Total_**	**−133.67** (**0.50**)	**−46.30** (**0.63**)	**−117.52** (**0.69**)	**−48.01** (**0.50**)

Values in parentheses are standard errors of the means. Explanation for the abbreviations can be found in materials and methods. ΔG_elec_ corresponds to sum of gas-phase electrostatic energy and polar solvation energy.

### Computational Analysis of Hot-Spot Interactions in D1 and D2

**Figure 2:**
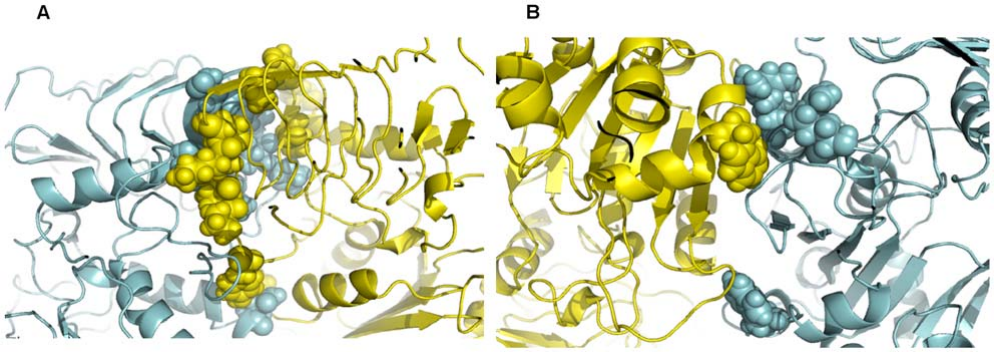
Snapshots of MD simulations from the final structures heterotetrameric. AGPase. MD analyses indicate the hot-spot residues (A) in D2 and (B) in D5. LS is cyan and SS is yellow in color. Hot-spots are shown in spheres.

In this study, the definition for hot spots is as follows: If a residue shows 3.0 kcal/mol energy drop in dimer formation compared to its subunit form (|ΔG_binding_|>3.0 kcal/mol), then it is considered as a hot spot. Hot-spot residues for D1 and D2 and their binding free energy components together with the standard deviations are shown in **Table 2** and **Table 3**, respectively. For a residue to be considered in interface its absolute SASA must decrease at least 1Å^2^ upon subunit complexation and it must satisfy this condition for at least 160 of the snapshots. Based on these requirements, a total of 79 (38 in LS and 41 in SS, data not shown) residues in D1 were classified to be part of interfaces. A total of 19 out of 79 interface residues (8 in LS and 11 in SS) in D1, are hot-spots. The hot-spot residues in LS are mostly non-polar in general with the exception of Asn^97^, Thr^328^, and His^342^. Seven of the hot-spots in SS for D1 are also non-polar, too. Residues SSLys^288^, SSTyr^308^, SSLys^313^ and SSThr^320^ make up the polar region in this interface. Overall interaction in the lateral dimer is mediated by amino acids that have hydrophobic side chain (**Figure 2A**).

**Table 2:** Free energy decomposition of hot spot residues in Dimer 1 (Values are in kcal/mol).

Residue	ΔE_ele_	ΔE_vdw_	ΔG_polar_	ΔG_non-polar_	ΔG_backbone_	ΔG_side-chain_	ΔG_total_
**LS**							
Asn97	−9.99±4.37	−4.00±1.20	11.06±4.61	−0.44±0.13	−0.57±0.44	−2.79±1.25	**−3.36**±1.12
Pro327	−0.50±0.40	−5.28±0.50	1.32±0.32	−0.57±0.03	−1.80±0.31	−3.24±0.47	**−5.03**±0.53
Thr328	−4.21±0.94	−2.23±0.58	3.35±0.61	−0.19±0.03	−2.26±0.57	−1.03±0.22	**−3.29**±0.57
Ile330	−2.99±1.18	−4.32±0.61	2.82±0.72	−0.38±0.03	−1.91±0.54	−2.97±0.35	**−4.88**±0.61
Ile335	−0.68±1.15	−4.13±0.74	0.29±0.67	−0.40±0.03	−1.73±0.42	−3.19±0.41	**−4.92**±0.56
Ile339	−0.43±0.47	−3.09±0.44	0.10±0.39	−0.24±0.03	−1.54±0.26	−2.12±0.42	**−3.66**±0.49
Ile340	−3.73±1.27	−3.27±0.54	4.01±0.82	−0.29±0.08	−1.54±0.46	−1.74±0.64	**−3.28**±0.96
His342	−8.47±1.34	2.34±0.76	7.72±0.86	−0.38±0.04	0.21±0.09	−3.27±0.60	**−3.48**±0.62
**SS**							
Met84	−0.57±1.19	−3.67±0.99	1.50±1.00	−0.52±0.13	−0.22±0.30	−3.03±1.08	**−3.25**±1.13
Lys288	−38.56±7.60	−0.47±0.70	36.09±6.67	−0.21±0.05	−0.44±0.17	−2.71±1.10	**−3.15**±1.13
Tyr308	−5.42±2.11	−6.63±0.99	6.12±1.32	−0.81±0.06	−1.99±0.88	−4.75±0.94	**−6.75**±1.16
Pro310	−3.24±0.71	−3.57±0.46	3.94±0.64	−0.33±0.04	−0.78±0.24	−2.41±0.42	**−3.19**±0.48
Pro311	1.59±0.82	−4.47±0.56	−0.50±0.60	−0.57±0.03	−0.97±0.40	−2.97±0.50	**−3.94**±0.68
Lys313	−102.96±5.58	−3.56±1.01	102.40±4.61	−0.65±0.04	−1.24±0.28	−3.54±1.57	**−4.77**±1.59
Met314	−3.70±1.23	−4.23±0.58	3.93±1.13	−0.40±0.04	−2.23±0.37	−2.18±0.52	**−4.41**±0.66
Val319	−3.17±1.00	−3.57±0.57	2.60±0.58	−0.32±0.02	−2.36±0.53	−2.11±0.33	**−4.47**±0.58
Thr320	−7.80±2.56	−2.21±0.44	6.49±1.75	−0.15±0.03	−2.19±0.35	−1.49±0.83	**−3.67**±0.96
Val323	−0.39±0.38	−2.78±0.38	0.07±0.30	−0.19±0.04	−1.55±0.19	−1.74±0.38	**−3.29**±0.43
Ile324	−5.92±0.89	−3.09±0.61	5.12±0.45	−0.29±0.03	−2.29±0.49	−1.89±0.31	**−4.18**±0.57

**Table 3:** Free energy decomposition of hot spot residues in Dimer 2 (Values are in kcal/mol).

Residue	ΔE_ele_	ΔE_vdw_	ΔG_polar_	ΔG_non-polar_	ΔG_backbone_	ΔG_side-chain_	ΔG_total_
**LS**							
Arg45	−89.00±14.39	0.08±0.78	83.81±11.20	−0.16±0.08	0.07±0.04	−5.35±3.14	**−5.28**±3.15
Arg88	−89.46±11.40	−4.15±0.81	90.33±8.50	−0.55±0.05	−0.18±0.08	−3.64±2.85	**−3.82**±2.86
Arg92	−88.88±3.83	−0.53±0.90	83.25±3.17	−0.21±0.04	−0.03±0.07	−6.34±1.03	**−6.37**±1.01
Trp135	−5.63±1.25	−4.44±0.63	6.20±0.82	−0.52±0.06	−0.11±0.05	−4.28±0.86	**−4.39**±0.88
**SS**							
Trp120	−5.46±1.09	−6.04±0.59	6.17±0.88	−0.62±0.03	−0.06±0.07	−5.88±0.76	**−5.95**±0.77

When we looked at the D2, we identified total of 53 amino acids (27 in LS and 26 in SS, data not shown) as interface residues. Number of hot-spots (five) in D2 is relatively less than the residues in D1. In contrast to D1 hot-spots, which are generally non-polar, there are three basic hot spot amino acids (Arg^45^, Arg^88^, Arg^92^ in LS) in this interface. The remaining two residues are Trp^135^ in LS and Trp^120^ in SS (**Figure 2B**). A recent MD study indicated that stable complexes prefer to use hydrophobic interactions rather than polar interactions [24]) in concordance with previous studies [25]–[28]. Further, the critical residues are found to be less mobile in the interfaces [29] contributing more to the stability. Here, we observe that D1, which is the lateral dimer, is more stable compared to D2 (longitudinal dimer) (see **Table 1**, the last row).

As can be seen from **Table 2**, Tyr^308^ in SS (in D1) shows the highest free energy difference with a |ΔG_binding_| value of 6.75 kcal/mol upon complexation. We see that favorable contributions to ΔG_binding_ for this residue are dominated by E_ele_ (–5.42 kcal/mol) and E_vdw_ (−6.63 kcal/mol). Indeed, several H-bonds are formed by Tyr^308^ and several polar residues (with the Thr^93^, Asn^97^ and Thr^320^ in LS). Tyr^308^ is also in close contact with non-polar residues, such as Pro^322^ in LS which account for the favorable van der Waals interactions. The unfavorable contribution of polar solvation energy (6.12 kcal/mol) comes from these interactions and it is observed to be compensated by the favorable electrostatic term. The backbone and side chain contributions to the total free energy are −1.99 and −4.75 kcal/mol, respectively for this residue. Pro^327^ in LS has the second highest |ΔG_binding_| energy difference with a value of 5.03 kcal/mol. It should be noted that this residue is highly conserved and makes van der Waals contacts with Gly^40^, Ala^41^, Ile^285^, Ile^324^ and the aromatic ring of Tyr^43^ in SS. These interactions explain the hydrophobic contribution of Pro^327^ to the total |ΔG_binding_|. The backbone and side chain contributions to the total free energy are −1.80 and −3.24 kcal/mol, respectively for Pro^327^.

**Figure 3:**
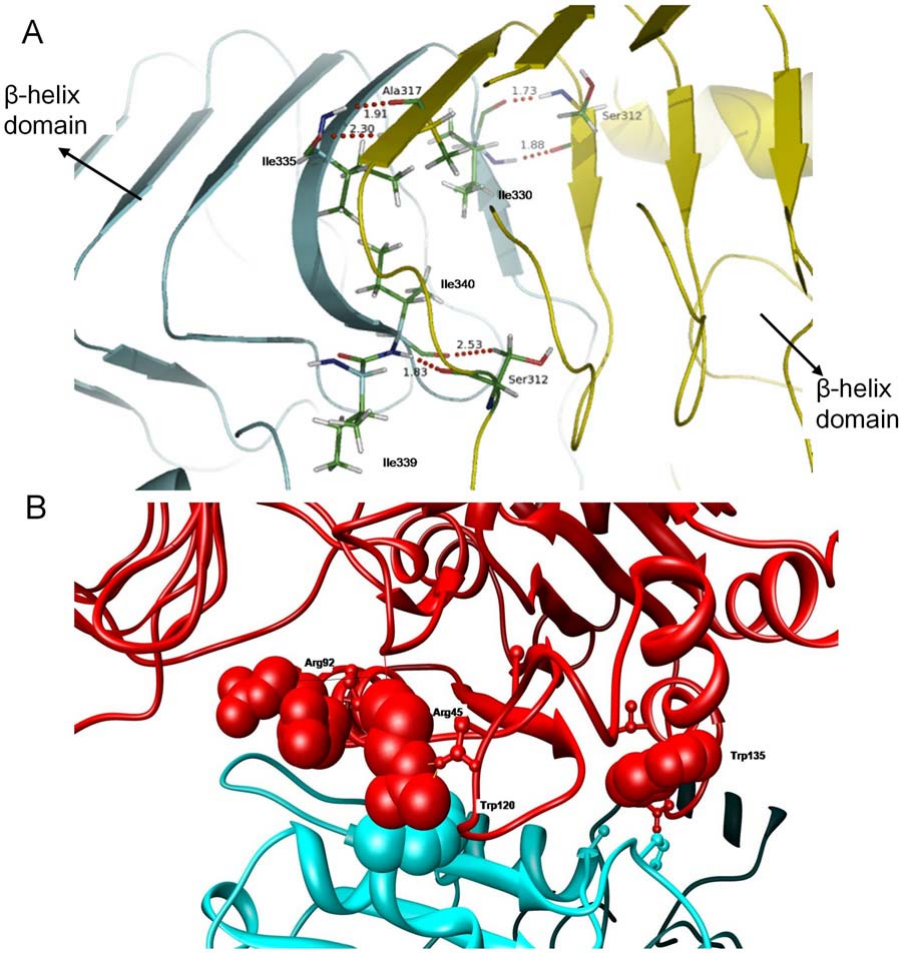
H-bonds between the SS and LS AGPase. (A) Snapshot showing the six H-bonds (red dashed lines) between Ile330-Ser312, Ile335-Ala317 and Ile340-Ser312 and their corresponding distances. These H-bonds are broken and reformed throughout the simulation. Ile^322^ is also illustrated in the picture. LS is shown cyan and SS is shown yellow in color. (B) Ribbon diagram of the interface region in the longitudinal dimer. Critical residues are highlighted.

Shown in **Figure 3A** are the three important isoleucine residues in LS, Ile^330^, Ile^335^ and Ile^340^ that constitute a hydrophobic core at the inner layer of β-helix domain. The bulky side-chain groups of these residues make strong hydrophobic interactions with each other as well as their counterparts in SS. In fact, favorable ΔG_binding_ for these amino acids are mainly driven by the van der Waals forces (see **Table 2**). E_ele_ terms on the other hand are canceled by the desolvation penalties during dimer formation. Also, noteworthy about Ile^330^, Ile^335^ and Ile^340^ is that they form total of six highly conserved H-bonds with Ser^322^, Ala^317^ and Ser^312^ in SS respectively (**Figure 3A**). Even though the effects of these H-bonds are counter balanced with the polar solvation terms, they contribute strongly to help the LS and SS β-helix domains to maintain their correct orientation relative to each other by providing structural constrains in subunit association. Modeled structure of LS reveals that side-chain of Ile^339^ is excluded from the hydrophobic core of β-helix domain. Instead this bulky group faces Ile^355^, another isoleucine whose side-chain is also excluded from the hydrophobic core, Thr^303^ and Lys^313^ in SS. Again E_vdw_ term plays a dominant role for the favorable energy state of this residue.

Lys^313^ in SS has a remarkable feature in terms of electrostatic and polar solvation energies. Upon complex formation this residue is surrounded by many non-polar amino acids such as Leu^302^, Ala^338^, Ile^339^ in LS and Leu^315^, Val^329^ in SS which are responsible for the high ΔG_polar_ term. However, it also contacts with LS Thr^303^ Gln^304^, Glu^305^ and polar groups and takes part in several H-bonds with these residues. These electrostatic interactions strongly favor the ΔG_binding_ for Lys^313^. It should be noted that, backbone free energy contributions of almost all residues are high (except Asn^97^, His^342^ in LS). This is especially important to decide whether the side chains or backbone interactions are important to define critical residues, hot spots, in AGPase complex.

As shown in **Table 3**, 80% of the hot-spots in D2 belong to LS in the longitudinal association. It is also worth mentioning that contributions of side-chain atoms in dimer stabilization are much higher than the backbone atoms in this group. This might suggest that longitudinal interactions are not as optimized as the lateral interactions and may further mean that even single alanine mutations on these residues can have deleterious effects in subunit-subunit interactions. We see that E_vdw_ term has no contribution for Arg^45^ stabilization in LS during dimerization (**Figure 3B**). Consequently, nearly all the contributions come from electrostatic interactions. This residue makes H-bonds with Ser^83^ and Glu^448^ in the LS and Glu^124^ in the SS. Although Arg^45^ is not surrounded with hydrophobic amino acids upon complexation it suffers from desolvation effects. One possible explanation for the high desolvation free energy might be that residues found in the close proximity of Arg^45^cannot sufficiently mimic the solvent environment in complex form. Trp^135^ in LS is enclosed by both polar and non-polar groups. Residues in the first group are Asn^142^ in LS, Asn^68^, Gln^100^ and Ser^101^ in SS which are the constituents of E_ele_. Second group of amino acids include Val^136^ in LS, Ala^70^ and Pro^102^ in SS (see **Figure 3B**). Contributions of Val^136^ and Ala^70^ to the E_vdw_ term might be smaller than Pro^102^ since the aromatic group of Trp^135^ can get involved in strong hydrophobic interactions with the side-chain of this residue.

### Comparison of Interactions between Dimers of SS and LS with the Crystal Structure of Homotetrameric SS

Several residues were reported to be critical in the crystal structure of homotetrameric SS by Jin et al [20]. To compare these amino acids with the corresponding residues in our AGPase model, free energy decomposition scheme was also applied to D3 and D4 (**Figure 1C**). **Table 4** and **Table 5** show interface amino acids in SS and their ΔG_binding_ values in our AGPase model and in the crystal structure of homotetrameric SS, respectively. All of the residues listed in **Table 4** were found to be part of interfaces according to our analysis. Four of the residues in the D3 (Tyr^308^, Pro^310^, Val^319^ and Ile^324^) and Trp^120^ in D4 were also classified as hot-spots in our AGPase model. All the other residues, except for the Glu^94^, also have negative ΔG_binding_ values which mean that they are stabilized upon complex formation. However, they were not considered as hot-spots since their change in ΔG_binding_ values according to free energy decomposition are higher than our cutoff value (−3.0 kcal/mol). We see that while the important amino acids reported by Jin et al. [20] have a total of −36.81 kcal/mol ΔG_binding_ energy in our model, they are less stabilized in the homotetrameric SS with a ΔG_binding_ value of −29.03 kcal/mol (**Table 5**). In other words, those residues are more stabilized if they interact with residues from LS instead of residues from SS. This is especially true for Tyr^308^, Lys^313^, Ile^324^ and Glu^124^. In addition, Glu^94^ has smaller positive ΔG_binding_ energy in our model. These results support the fact that a SS chain prefers to interact with a LS instead of another SS in terms of thermodynamic stability.

**Table 4:** ΔG_binding_ values of important residues in SS.

	D1									D2				
**Residue**	Y308	L309	P310	K313	V319	D321	S322	I324	Y363	E90	E94	Q100	W120	E124
**ΔG_binding_**	−6.75	−1.67	−3.19	−4.77	−4.47	−1.29	−1.97	−4.18	−0.61	−1.09	2.17	−1.97	−5.95	−1.07
**SEM** §	1.16	0.54	0.48	1.59	0.58	0.59	0.58	0.57	0.34	1.80	0.60	0.84	0.77	1.53

**§:** Standard error of mean. These residues are reported by Jin et al (19) in our AGPase model. Values are in kcal/mol. Note that interface residues in A and C chains are not listed since these chains are occupied with LSs in our AGPase model. Results were obtained from the free energy decomposition of LS-SS interaction (D1 and D2 in Fig. 1b).

**Table 5:** ΔG_binding_ values of important residues in single chain SS.

	D1									D2				
**Residue**	Y308	L309	P310	K313	V319	D321	S322	I324	Y363	E90	E94	Q100	W120	E124
**ΔG_binding_**	−2.48	−1.71	−2.94	−1.67	−4.20	−2.69	−3.07	−3.58	−0.98	−0.74	3.30	−2.79	−5.16	−0.32
**SEM** §	0.64	0.63	0.49	1.07	0.55	1.01	0.55	0.70	0.39	0.97	0.49	1.05	0.81	1.14

**§:** Standard error of mean.

### Analysis of Hot-Spots by Yeast Two Hybrid Method and Bacterial Complementation Assays

**Figure 4:**
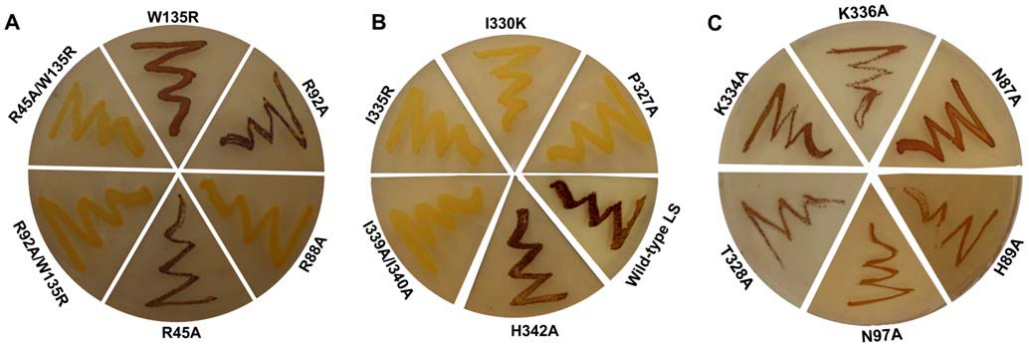
Bacterial complementation assay using various mutants of the LS and the wildtype SS. Iodine vapor staining of *E. coli* AC70R1-504 (glgC^−^) containing wild-type SS potato AGPase (pML10) and mutant or wild-type LS AGPase (pML7). The plate was streaked from a single colony of each strain onto a Kornberg's 2% glucose enriched plate and incubated overnight at 37°C. From A to C plates containing various mutants of the LS and the wildtype potato AGPases.

It has been previously shown that the expression of the cDNA sequences of the potato tuber LS and SS subunits yielded a functional heterotetrameric enzyme capable of complementing a mutation in the single AGPase (*glg*C) structural gene of *Escherichia coli* [13]. This heterologous complementation provides a powerful genetic approach to obtain biochemical information on the specific roles of the LS and the SS in enzyme function [13],[30]. We performed site-directed mutagenesis experiments based on the results of the MM-GBSA method. Computationally identified hot spots of the LS were mutated and then expressed in an *E. coli glg*C^−^ (containing pML10). The ability of LS mutants to form a functional heterotetrameric AGPase was assessed by exposing mutant colonies to I_2_ vapor to monitor the glycogen accumulation. The residues of the LS listed in **Tables 2** **and** **3** were mutated. Cells carrying the LS Pro327Ala, Thr328Ala, Ile330Lys, Ile335Arg, or Ile339Ala/Ile340Ala mutants within the αβ domain along with the wildtype (WT) SS displayed an impaired glycogen accumulation compared to cells co-expressing WT potato AGPase genes (**Table 6** and **Figure 4A, C**). On the other hand, cells expressing the LSHis342Ala, LSAsn97Ala and WT SS demonstrated a comparable glycogen accumulation compared with cells co-expressing potato AGPase genes (**Figure 4A**). As a control, Lys^334^ (ΔG_binding_ = −2.19 kcal/mol) and Lys^336^ (ΔG_binding_ = −2.83 kcal/mol) adjacent to the Ile^335^ were replaced with Ala. These LS mutants were transformed into *E. coli glgC* ^−^ cell lines containing the WT SS. Cells were exposed to iodine staining to see the effect of mutation on the heterotetrameric assemblies. As seen in **Figure 4C** cells harboring those mutants were stained with iodine. These results suggested that altering amino acid residues of hot spots of the LS disturbed the heterotetrameric AGPase assemblies in *E. coli*. Our results are in agreement with previously reported data where they showed that lateral interaction is mainly mediated by the hydrophobic amino acids in homotetrameric enzymes of the potato SS and *Agrobacterium* AGPases within the αβ domain of AGPase [20],[31]. Also, changing the size and polarity of any amino acids in the interacting region of LS disturbed heterotetrameric structure of potato AGPase in *E. coli*. For example when Ile^339^, and Ile^340^ were changed to Ala (smaller R-side chain), there were no heterotetrameric assemblies between the potato LS and SS AGPase subunits in *E. coli* (**Figure 4A**). Similarly mutating the positions at Ile^330^ to Lys and Ile^335^ to Arg, which have different charge, again disturbed heterotetrameric assembly in *E. coli*.

**Table 6:** Functional analysis of selected hot-spot residues with comparison to backbone and total ΔG_binding_ energy values.

LS Residue	ΔG_backbone_	ΔG_total_	Mutation	Iodine staining level
**D1**			WT	++
Pro327	−1.80	−5.03	Pro327Ala	−
Ile330	−1.91	−4.88	Ile330Lys	−
Ile335	−1.73	−4.92	Ile335Arg	−
Ile339	−1.54	−3.66	Ile339Ala/Ile340Ala	−
Ile340	−1.54	−3.28		
His342	−0.21	−3.48	His342Ala	++
**D2**				
Arg45	0.07	−5.28	Arg45Ala	++
Arg88	−0.18	−3.82	Arg88Ala	−
Arg92	−0.03	−6.37	Arg92Ala	++
Trp135	−0.11	−4.39	Trp135Arg	+
			Arg45Ala/Trp135Arg	−
			Arg92Ala/Trp135Arg	−

**Figure 5:**
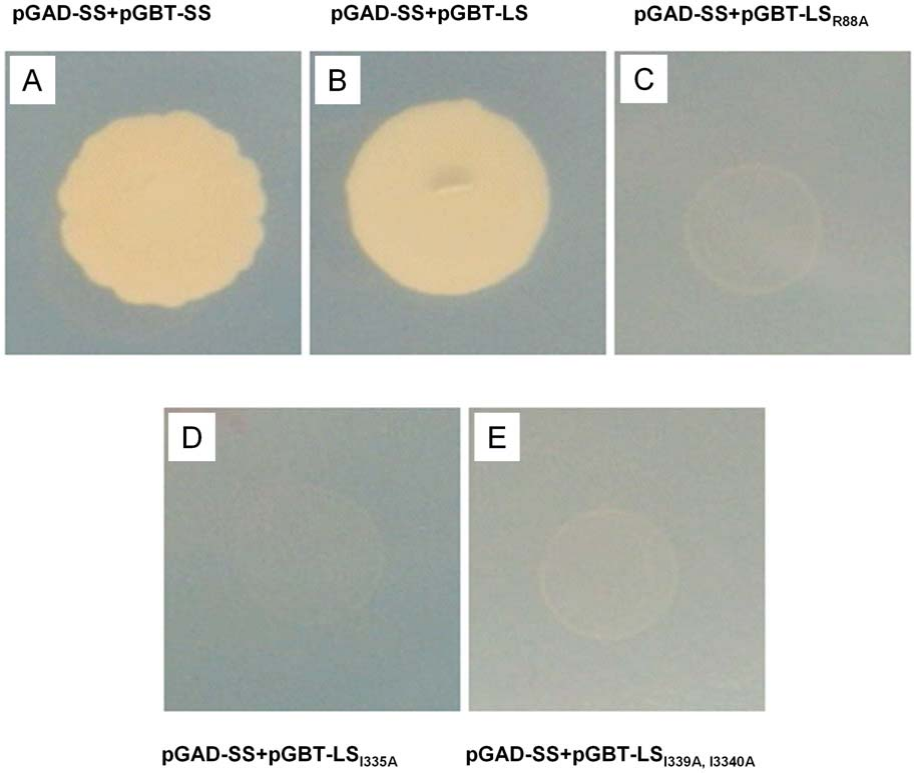
Analysis of the interaction between the potato wildtype/mutants LS and the potato SS AGPase by yeast two hybrid. The interaction between (A) SSWT and SSWT; (B) SSWT and LSWT; (C) SSWT and LSR88A; (D) SSWT and LSI330A; (E) SSWT and LSI339A,I330A vector. AH109 yeast cells expressing the designated plasmids are selected on a synthetic growth medium without Leu and Trp. Selections for interactions were carried out in the absence of Leu, Trp, and His.

Next, we investigated the role of amino acids (Arg^45^, Arg^88^, Arg^92^ and Trp^135^) of the potato AGPase LS in longitudinal interaction with the potato AGPase SS by bacterial complementation and yeast two hybrid assays. Residues, Arg^45^, Arg^88^, and Arg^92^ were mutated to the Ala whereas Trp^135^ was mutated to Arg by site-directed mutagenesis in pML7 vector. Mutants were transformed into *E. coli glg*C^−^ (with the pML10). Only the LSArg88Ala mutants have glycogen deficient phenotype and they were unable to complement *glg*C^−^ gene compared to cells containing wildtype AGPase genes (**Table 6**, **Figure 4b**). To see if Arg^88^ is solely responsible for the interaction with SS, as a control we replaced adjacent amino acids Asn^87^ (ΔG_binding_ = −.123 kcal/mol) and His^89^ (ΔG_binding_ = −0.11 kcal/mol) to Ala. Bacterial complementation result indicated that cells harboring mutant LS constructs can complement *glg*C^−^ in *E. coli* (**Figure 4C**). The LSArg88Ala mutant colonies were unable to grow on the selective medium compared with cells carrying WT genes in yeast two hybrid experiments(**Figure 5**). Bacterial cells carrying LSArg45Ala or LSArg92Ala mutants displayed an identical phenotype with the cells containing WT LS and WT SS in *E. coli* (**Figure 4B**). Moreover, cells co-expressing the LSTrp135Arg mutant and WT SS demonstrated moderate staining compared to cells expressing WT genes (**Figure 4B**).

**Figure 6:**
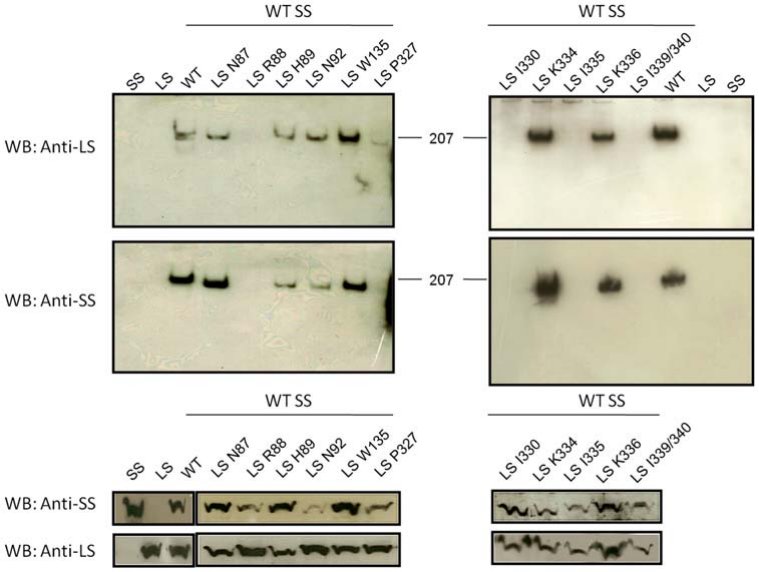
Heterotetrameric assembly of mutants and wildtype potato AGPases. Western Blot analysis of various mutants of the LS and wild type SS. Top two panels belong native gels. 10 µg of total protein from crude extract were loaded on 3–13% native gradient gel and followed by western blot using anti-LS and anti-SS antibodies. Bottom two panels show western blot results from 10% SDS-PAGE using anti-LS and anti-SS antibodies.

To see the direct effects of mutations on heterotetrameric assemblies, WT and mutant constructs (of LS) were expressed with the WT SS in *E. coli glgC* ^−^ using IPTG and nalidixic acid (see Materials and Methods). Then, cells were disrupted in the presence of protease inhibitors and 10 µg of total protein of each sample were subjected to 10% SDS-PAGE followed by Western Blot analysis using anti-LS and anti-SS antibodies. As shown in **Figure 6** all the LS mutants and SS proteins were detected around 50 kDa as expected in the cell-free extract. Then, crude extract of these samples were analyzed by 3–13% gradient native PAGE to determine if these mutated LS subunits were able to assemble with their counterpart WT subunits to form the oligomeric structure. The expressed LS and SS by themselves can not be seen as homotetrameric structures in native gel compared with when both subunits are together (**Figure 6**, look for SS only, LS only and WT AGPase). It is worth to note that, although SS homotetramer can form *in vitro*, they are not stable enough to be seen by native gel. The WT AGPase was detected around 200 kDa compared with trimeric BSA control. The LS mutants of Arg88Ala, Pro327Ala, Ile330Lys, Ile335Arg, Ile339Ala/Ile340Ala were unable to form heterotetrameric structures. If these amino acids of the LS directly contribute to the interaction with the SS, one would expect to see nearby amino acids not interfering with heterotetrameric interaction. Therefore, we have randomly changed adjacent amino acids of Arg^88^ and Ile^335^ and analyzed by the native gel. Replacement of the both Asn^87^ and His^89^ to Ala did not effect heterotetrameric formation (**Figure 6**). Likewise, changing amino acids of Lys^334^ and Lys^336^ into Ala resulted in heterotetrameric structure (**Figure 6**). These results are in agreement with staining data that indicated that we successfully identified amino acid residues of the LS that mediates interaction with the SS.

When we analyzed the backbone energy contribution of Arg^45^, Arg^88^, Arg^92^, and Trp^135^ of the LS with interaction of the SS, Arg^88^ had the highest backbone energy (**Table 3** **and** **4**). Then, we hypothesized that these residues themselves may not be enough to interrupt the heterotetrameric assembly and we subsequently generated double mutants. The LS double mutants, Trp135Arg/Arg45Ala and Trp135Arg/Arg92Ala, were transformed into the *E. coli glg*C^−^ containing the pML10 (WT SS). As seen in **Figure 4B**, both LS mutants were not able to complement *glg*C^−^ gene in *E. coli* and in turn glycogen production. These results point out that the backbone energy of these residues showed an additive effect when they combined and caused disruption of the heterotetrameric assemblies.

## Discussion

**Figure 7:**
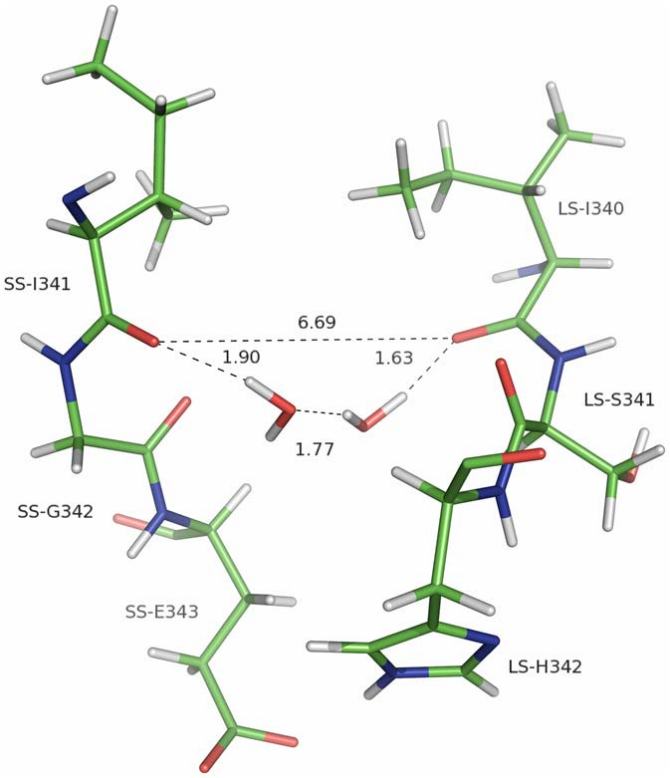
Presences of water molecule between the H-bonds. Hydrogen bonding network between SS-I341 and LS-I340 provided by two water molecules trapped between the interfaces of D1.

**Figure 8:**
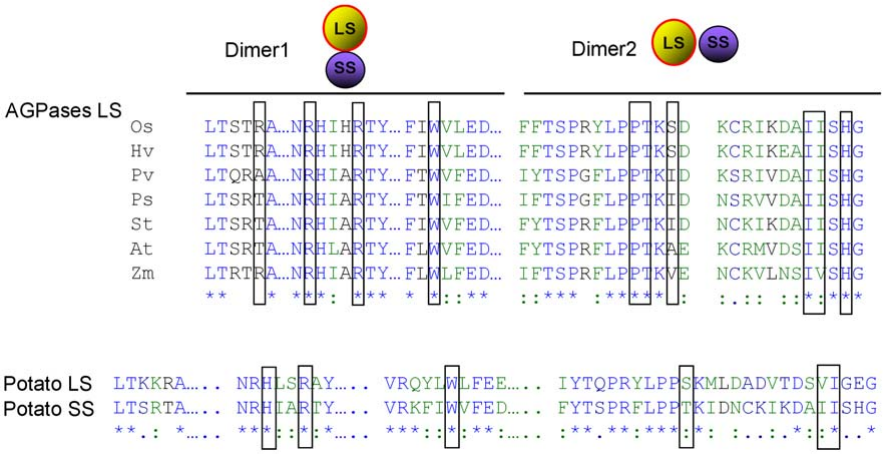
Aligment of the potato large subunit AGPase with various plant LS and with potato SS. (A) Primary amino acids sequences alignment of various LS AGPase. Boxed amino acids play direct role with interaction of the SS AGPase. OS, *Oryaza sativa*; Hv, *Hordeum vulgare*; Pv, *Phaseolus vulgaris*; St, *Solanum tuberosum*, At, *Arabidopsis thaliana*; and Zm, *Zea mays*. (B) Comparison of primary amino acid sequence alignment of the potato AGPase LS and SS. Box indicated conserved amino acid residues that are important for the subunit-subunit interaction.

Plant AGPases contain two different subunits encoded by two different genes [6],[13]. Recently, the homotetrameric structure of the potato tuber AGPase SS and *Agrobacterium* AGPase have been reported [20],[31]. These structures are far from the native structure of the heterotetrameric plant AGPases. On the other hand, having these structures allowed us to model first the LS of potato AGPase and then the heterotetrameric structure [23]. In this study, we have used our modeled structure to map the energetic contributions of the exact amino acids that mediate heterotetrameric assembly of potato AGPase by using both computational and experimental methods. It should be noted that the MM-GBSA method used in this study treats the explicit water molecules as a continuum environment. Such an assumption may result in miscalculation of short-range interactions, temperature and pressure effects. Also, it neglects the frictional forces between water and protein observed during the simulation [32],[33]. Water molecules can also act as a stabilizing factor between two atoms of different residues that would otherwise affect the interaction negatively or may even provide an interaction between two residues by a hydrogen bonding network. This is especially true for water molecules that are trapped between the interfaces of interacting partners. Shown in **Figure 7** is a hydrogen bonding network between the backbone oxygen atoms of LS-Ile^340^ and SS-Ile^324^ provided by two water molecules in D1. These water molecules form several hydrogen bonds with the Ile residues separately during the simulation and the same network as in **Figure 7** in 5 ns of the snapshots. In addition, the free energy decomposition scheme may fail to calculate the precise contribution of individual amino acids to binding free energy since these energies are obtained by addition and subtraction of large numeric values leading to high standard errors. This effect can be observed in the case of ΔE_ele_ and ΔG_polar_ values for Lys^288^, Lys^313^ in SS (D1) and Arg^45^, Arg^88^ and Arg^92^ in LS (D2). Based on these considerations, although our MM-GBSA calculations may not be perfect, they are fairly consistent with the experimental results. Total of 79 (38 in LS and 41 in SS) residues in lateral interaction and 53 residues (27 in LS and 26 in SS) in longitudinal interactions were classified to be part of interfaces. Free energy decomposition scheme was applied to identify the critical residues in the LS-SS interfaces. In both cases, residues that showed 3.0 kcal/mol energy drop upon complexation of the LS and the SS were defined as hot-spots. A total of 19 out of 79 interface residues (8 in LS and 11 in SS) and 5 of 41 residues (4 in LS and one in SS) were accepted as hot-spots. Interestingly, the identified hot-spot residues in LS are highly conserved among different species (**Figure 8A**). Furthermore, Greene and Hannah [34] have identified an amino acid residue (His^333^) from the maize endosperm LS AGPase that participates in interactions with the SS. Our analysis of interface residues of potato LS indicated that Tyr^275^ (corresponding to maize His^333^ LS AGPase) is not involved in interaction. This specific residue may be solely responsible for heat stability rather than any interaction between the subunits. There are many studies performed to understand the subunit-subunit interaction of the AGPase subunits mainly carrying out domain swap between the different species subunits [19],[35],[36]. Those results mainly indicated that domains from different species are compatible and chimeric subunits render a functional enzyme and, in turn, heterotetrameric AGPases. As seen in **Figure 8A**, amino acids in D1 and D2 are well conserved and domain swap experiments will not abolish interaction of the subunits. Also, it is interesting to note that many of those residues are conserved in the potato SS AGPase as well (**Figure 8B**).

As discussed above, the heterotetrameric structure consists of lateral and longitudinal interactions. The LS residues, Asn^97^, Pro^327^, Thr^328^, Ile^330^, Ile^335^, Ile^339^, Ile^340^, and His^342^, were involved in lateral LS-SS interaction whereas the LS residues of Arg^45^, Arg^88^, Arg^92^, and Trp^135^ were involved in longitudinal LS-SS association. These residues were mutated and the effect of these mutations on the interactions of LS and SS were characterized *in vivo* using the yeast two-hybrid method and the bacterial complementation assay. Mutating the LS residues at position 88, 327, 330, 335 and 339/340 abolished the interaction between the LS and the SS subunits. On the other hand, the LS residues at position 45, 92, 97, 328 and 342 have no significant contribution to the subunit-subunit interactions. When we compared the MM-GBSA results with the experimental data, we made a very interesting and insightful observation: Residues with favorable backbone free energy terms actually correspond to critical residues (**Table 6**). When candidate hotspots compared with respect to backbone free energy decomposition, His^342^ (−0.21 kcal/mol) and Asn^97^ (−0.57 kcal/mol) residues have an insignificant effect in the lateral interaction. Changing the LS residues at position 327, 330, 335 and 339/340 abolished the interaction between LS and SS and inhibited the glycogen synthesis; however, His342Ala mutants were able to synthesize glycogen. Experimental results confirmed the computational analysis using MM-GBSA method, and exhibited a remarkable concordance with backbone **ΔG_binding_** energy values instead of side chain or total **ΔG_binding_**.

We have previously mentioned that Ile^339^ side-chain is excluded from the inner part of the β-helix domain and van der Waals energies (−3.09 kcal/mol) have a dominant contribution to the favorable ΔG_binding_ of this residue which is mainly due to the interactions with Ile^338^ of the SS. Mutation of this residue to Ala will not only decrease the E_vdw_ term, but may also force the side-chain of alanine to be included in the inner layer of β-helix domain. This is highly possible since Ile^340^, whose side-chain is involved in the inner layer, is mutated to alanine at the same time and the residue at position 321 of the SS is also an alanine. Such an inclusion will certainly result in steric clashes between the side-chains and disturb the interface structure of β-helix domain where it makes important interactions with SS. In other words, this mutation can affect the interactions both energetically and structurally.

Among the four candidate hotspot residues involved in longitudinal LS-SS association, the backbone free energy decomposition of Arg^88^ residue has the highest value and mutant cells were defective in glycogen synthesis. The backbone free energy decomposition of Trp^135^ (−0.11 kcal/mol) residue is less favorable than Arg^88^ (−0.18 kcal/mol) but more than Arg^45^ (0.07 kcal/mol), and Arg^92^ (−0.03 kcal/mol). Consistently, glycogen synthesis was reduced in cells expressing Trp135Arg when compared with Arg45Ala and Arg92Ala. Although mutation at residues of Arg^45^ and Arg^92^ displayed no effect on the subunit-subunit interactions, the coupling of these residues with Trp^135^ abolished the heterotetrameric structure formation, hence, inhibited the glycogen synthesis suggesting the importance of each identified residue and cooperative effect of these residues.

Thr^328^ and Ile^330^ double mutation to alanine seems to have an effect on D1 formation according to yeast two hybrid result (data is not shown) because these residues account for a total of −8.17 kcal/mol. We see in **Table 2** that side-chain of Thr^328^ has a minor contribution (−1.03 kcal/mol) to ΔG_binding_ compared to its backbone (−2.26 kcal/mol). Having high backbone energy increases the chance of an alanine mutation to disturb the interactions between the subunits. In addition, an alanine mutation at this position might result in a decrease in the electrostatic interactions, which is −4.21 kcal/mol. Thus, these balancing changes decrease the overall effect of the alanine mutation. It is obvious that an alanine mutation at Ile^330^ position will certainly decrease the van der Waals effects (−4.21 kcal/mol) of this residue. As previously mentioned Ile^330^ makes two H-bonds with Ala^317^ in SS which accounts for the −2.99 kcal/mol electrostatic interactions.

### Conclusion

The data presented in this paper allow us to reach the following conclusions. First, critical amino acid residues of the potato LS AGPase subunit that interact with SS subunit were identified using MM-GBSA and experimental methods. Lateral interaction between the LS and SS subunits was mainly mediated by the hydrophobic amino acids as shown previously for homotetrameric AGPase . For the first time we have shown the amino acids of the LS subunit that are important for such interactions The amino acids Asn^97^, Pro^327^, Ile^330^, Ile^335^, Ile^339^, Ile^340^, and His^342^ are critical for the interaction with the SS of AGPase. Longitudinal interaction by the LS AGPase with the SS subunit is mediated by the Arg^42^, Arg^88^, Arg^92^, and Trp^135^. Second, we found that dimer 1 is much more stable compared with dimer 2 due to the hydrophobic interaction in dimer 1. Finally, backbone energy is an important deterministic parameter for the protein-protein interaction.

## Materials and Methods

### Structure Prediction for Potato AGPase Large Subunit and Model Proposition for the Native AGPase

Potato tuber AGPase large and small subunits share 53% sequence identity according to the CLUSTALW [37] results. Such a high sequence identity between the subunits allows us to model the potato LS AGPase structure using the homology modeling. In our previous study [23], we have predicted the three dimensional structure of the LS using the SWISS-Model homology modeling server. Then, we proposed three possible models for the native heterotetrameric AGPase based on the crystal structure of homotetrameric SS using MM-PBSA [23]. A schematic presentation of a proposed heterotetrameric structure can be seen in **Figure 1A**. The lateral and longitudinal dimers of LS and SS are labeled as D1 and D2, respectively (**Figure 1B**).

### Molecular Dynamics Simulations

Explicit solvent molecular dynamics simulations for the representative structures of the native AGPase, D1 and D2, were performed using NAMD software [38] with parm96 force field [39]. Starting structures were solvated in rectangular boxes of TIP3P [40] water molecules. Distances between the edge of the boxes and the closest atom of the solutes were adjusted to at least 10 Å. Counter ions were added, 10 Na^+^ atoms, in order to neutralize the systems. All the histidine residues were charged as +1 at their Nε atoms in order to establish unity. Particle Mesh Ewald (PME) method [41] was used to treat the long range electrostatic interactions and a direct space non-bonded cut off value was taken as 9 Å. Water molecules and the hydrogen atoms were constrained by applying the SHAKE algorithm [42]. Langevin piston Nose-Hoover method [43], as implemented in NAMD, was used to keep the pressure of the systems constant together with the periodic boundary conditions (PBC). Time step of the simulations were 2 fs. Systems were first minimized for 10^4^ steps using conjugate gradient method and keeping the backbone atoms of the solute atoms fixed. Minimization was completed by an additional 10^4^ steps with all the atoms relaxed to remove the bad contacts. The systems were then gradually heated from 0 K to 300 K in 150 ps using canonical ensembles (NVT) during which the Cα atoms of the solutes were restrained by applying 2 kcal mol^−1^ Å^−2^ force constants. Subsequent shift into isothermal-isobaric (NPT) ensembles was done and harmonic restraints on the Cα atoms were gradually removed in 80 ps after which the systems were equilibrated with an additional 100 ps. NPT simulations were performed for 8 ns at 300 K from which the last 4 ns was used to extract the snapshots with 20 ps time intervals. The 200 snapshots were then used for interface residue identification and binding free energy calculations together with free energy decomposition scheme (see ref [23]).

### Identification of Interface Residues

Snapshots are taken from the last 4 ns of the simulations (200 snapshots with 20 ps intervals). Interface residues were determined using NACCESS [44] based on the implementation of Lee and Richards method. Calculations were performed for each of the complex and subunits separately excluding the hydrogen atoms. As probe radius values for the calculation of solvent accessible surface area (SASA), we have used 1.4 Å together with a z-slice value of 0.05 Å. Residues that showed 1 Å^2^ decrease in their SASA upon complex formation were considered as part of the interface. These amino acids were then further screened by an additional criterion to eliminate the pseudo interface residues. Residues that satisfy the above condition for at least 80% of the last 6 ns simulation time (160 snapshots) were treated as the true interface amino acids.

### MM-GBSA Analysis

In this study, MM-GBSA [45],[46] method was mainly employed to calculate the binding free energy of molecules in an equilibrium state. In this approach, binding free energy of a complex is calculated by taking snapshots from a molecular dynamics trajectory and computing the average energy of these snapshots according to the formula in Eq (1);
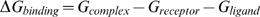
(1)where G_complex_, G_receptor_, G_ligand_ are the energies of the complex, receptor and ligand respectively. Each term on the right hand side of Eq 1 can be represented as shown in the following equation:
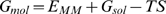
(2)where E_MM_ is the total mechanical energy of the molecule in gas phase, G_sol_ is the solvation free energy and TS is the entropic term. Each term in Eq (2) can be written as follows:
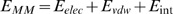
(3)where E_MM_ represents the bonded and non-bonded interactions as a sum of electrostatic (columbic), van der Waals (Lennard-Jones) and internal strain (bonds, angles and dihedrals) energies. This term is calculated by classical molecular-mechanics methods using standard force fields such as *parm96* force field [39]. Solvation free energy of a molecule is calculated as the sum of a polar and a non-polar term:
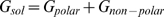
(4)where electrostatic contribution to the solvation energy (G_polar_) is computed in a continuum solvent environment by using the GBSA method. Non-polar solvation energy (G_non-polar_), which is considered to be the sum of a solute-solvent van der Waals interaction and solvent-solvent cavity formation energy, is approximated by using an empirical formula such as G_non-polar_ = α×SASA. According to this formula, non-polar solvation energy of a molecule is proportional to the solvent accessible surface area (SASA) of that molecule in a solvent, where α was taken as 0.005 kcal•Å^−2^ [47],[48]. The entropic term in Eq (2) is considered as the summation of vibrational, rotational and translational contributions where vibrational term can be calculated by normal-mode analysis or quasi-harmonic analysis:
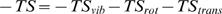
(5)The entropic term is found to be much smaller than the other two terms (in Eq. 2) in many applications of estimating *relative* binding free energies [46]. Since the calculation of entropic contribution is computationally expensive, this term can be omitted if qualitative results, rather than quantitative, are considered to be more important. This is also true for different ligands that show similar binding affinities and modes for a given receptor [49],[50]. However, neglect of entropic terms may lead to miscalculation of binding free energy, hence individual contributions of amino acids to total binding energy, if they show significant conformational change upon complex formation. In our study, this issue is more important for hot-spots in D2 (those found in β-helix domain) compared with the hot-spots in D1 since they are relatively more flexible in separate receptor (LS) and ligand (SS) forms. The last 4 ns of the simulations for both lateral and longitudinal dimeric interactions between the LS and SS pairs were analyzed by MM-GBSA method as implemented in AMBER8 package [51] (with igb = 2) with the modified Bondi radii (mbondi2) [47] which is appropriate for macromolecules such as proteins. The trajectories were post processed in order to strip off the water molecules and counter ions before the calculations. 200 snapshots with 20 ps intervals were extracted for each complex, receptor and ligand structures from single trajectories. We analyzed the autocorrelation functions of effective free energies and found that the correlations drop to 0.1 in 20 ps (**see ref** [23]).

In all the calculations the LS was treated as the receptor and the SS as the ligand. Gas phase energies (E_MM_) of the proteins were calculated by the SANDER module applying no cutoff value for non-bonded interactions. Dielectric constants for the solute and solvent were taken as 1 and 80, respectively; and the solvent probe radius was adjusted to 1.4 Å. Residues in interfaces of the subunits that showed at least 3 kcal/mol energy decrease, upon complexation, according to the per-residue free energy decomposition were considered as hot-spots.

### Cloning and Site Directed Mutagenesis of Large and Small Subunits

The cDNAs of potato AGPase LS and SS were PCR amplified using pML7 and pML10 plasmids as template, respectively. The restriction sites, NcoI and BamHI, were introduced using primers at **Table 7**. Then, the PCR products were subjected to restriction enzymes and ligated into pGADT7 and pGBKT7 vectors to construct pGAD-SS and pGBKT7-LS plasmids for the yeast two-hybrid assay. *E. coli* DH5α strain was used during the manipulation of plasmids. For bacterial complementation assays, plasmids pML7 and pML10 were used.

**Table 7:** Oligonucleotide primers used for amplification of the LS cDNA and generation of site-directed mutations.

PCR	Primer Sequence
**Cloning**	LS-F: 5′- AGCGCCGCCATGGTGATCACTACTGAAAATGACACA-3′
	LS-R: 5′- GATCCCCGGGAATTCTCATATGACTGTTCCATCTCTAATTG-3′
	SS-F: 5′-CATATGGCCATGGCTGTTTCTGATTCGCAGAATTC-3′
	SS-R: 5′-AGCTCGATGGATCCCTCAGATGATGATTCCACTTGG-3′
**SDM**	LSR45A-F: 5′- GTTATTCCCACTTACAAGTGCAACTGCAACCCCTGCTG-3′
	LSR45A-R: 5′- CAGCAGGGGTTGCAGTTGCACTTGTAAGTGGGAATAAC -3′
	LSN87A-F: 5′-CAATTCTGCTCCCCTGGCTCGTCACATTGCTCG-3′
	LSN87A-R: 5′-CGAGCAATGTGACGAGCCAGGGGAGCAGAATTG-3′
	LSR88A-F: 5′-GTACAATTCTGCTCCCCTGAATGCTCACATTGCTCGAACATAT-3′
	LSR88A-R: 5′-ATATGTTCGAGCAATGTGAGCATTCAGGGGAGCAGAATTGTAC-3′
	LSH89A-F: 5′-CCCCTGAATCGTGCCATTGCTCGAAC-3′
	LSH89A-R: 5′-GTTCGAGCAATGGCACGATTCAGGGG-3′
	LSR92A-F: 5′-CTGAATCGTCACATTGCTGCAACATATTTTGGCAATGGTG-3′
	LSR92A-R: 5′-CACCATTGCCAAAATATGTTGCAGCAATGTGACGATTCAG -3′
	LSN102A-F: 5′-CTCGAACATATTTTGGCGCTGGTGTGAGCTTTGGAG-3′
	LSN102A-R: 5′-CTCCAAAGCTCACACCAGCGCCAAAATATGTTCGAG-3′
	LSW135A-F: 5′-GATGCTGTTAGAAAATTTATAGCGGTTTTTGAGGACGCTAAG-3′
	LSW135A-R: 5′-CTTAGCGTCCTCAAAAACCGCTATAAATTTTCTAACAGCATC-3′
	LSW135R-F: 5′-GATGCTGTTAGAAAATTTATACGGGTTTTTGAGGACGCTAAG-3′
	LSW135R-R: 5′-CTTAGCGTCCTCAAAAACCCGTATAAATTTTCTAACAGCATC-3′
	LSP327A-F: 5′-TACACATCTCCTAGGTTCCTTCCAGCAACCAAGATAGACAATTGC-3′
	LSP327A-R: 5′-GCAATTGTCTATCTTGGTTGCTGGAAGGAACCTAGGAGATGTGTA-3′
	LSI330K-F: 5′-AGGTTCCTTCCACCAACCAAGAAAGACAATTGCAAGATTAAGG-3′
	LSI330K-R: 5′-CCTTAATCTTGCAATTGTCTTTCTTGGTTGGTGGAAGGAACCT-3′
	LSK334A-F: 5′-CCAACCAAGATAGACAATTGCGCGATTAAGGATGCCATAATCTC-3′
	LSK334A-R: 5′-GAGATTATGGCATCCTTAATCGCGCAATTGTCTATCTTGGTTGG-3′
	LSI335R-F: 5′-CAAGATAGACAATTGCAAGAGGAAGGATGCCATAATCTCTCATGGATG-3′
	LSK336A-F: 5′-GATAGACAATTGCAAGATTGCGGATGCCATAATCTCTCATG-3′
	LSK336A-R: 5′-CATGAGAGATTATGGCATCCGCAATCTTGCAATTGTCTATC-3′
	LSH342A-F: 5′-GGATGCCATAATCTCTGCTGGATGTTTCTTGCGAG-3′
	LST328A/I330A-F: 5′-CATCTCCTAGGTTCCTCCTTCCACCAGCCAAGGCAGACAATTGCAAGATTAAG-3′
	LSI339A/I330A-F:5′-GACAATTGCAAGATTAAGGATGCCGCAGCCTCTCATGGATGTTTCTTGCGAGATTGTTC-3′

Underlined and bold nucleotides indicate the nucleotides used to replace the wild-type amino acid. F, forward and R, antisense.

Site-directed mutations of the specified hot spot residues were introduced to potato AGPase LS by PCR. Plasmids pML7, pGBT7K-LS, or pGAD-SS were used as template. PCR reaction was performed in a total volume of 50 µl containing approximately 50 ng of plasmid samples, 20 pmol of each primer, 0.2 mM dNTPs, and 2.5 unit Dream Taq DNA polymerase (MBI Fermentas) with appropriate primers indicated at Table I. Conditions for the 18 cycles of amplification reaction were 95°C for 30 s, 50°C for 30 s and 68°C for 14 min. Before the first cycle reaction mixtures were kept at 95°C for 4 min and at the end of the 18^th^ cycle an additional 68°C extension period was applied for 10 min. Samples were then treated with DpnI restriction enzyme to remove the template DNA and transformed into *E .coli*. Transformed cells were seeded and selected on appropriate selective medium. The presence of the specific mutations was verified by DNA sequencing throughout Burc Laboratory (Istanbul, Turkey).

### Yeast Manipulations

Yeast-two hybrid assays were performed as described previously [23]. Briefly, the constructs containing wildtype (WT) or mutant LS were sequentially transformed into the cells as in the following procedure. First, pGAD-SS was transformed into AH109 cells. Transformed cells were plated on SD/-Leu medium. A single colony was inoculated in liquid SD/-Leu medium for competent cell preparation. Then, constructs that contain the WT or mutant LS were transferred into AH109/pGAD-SS cells. Transformed cells were seeded onto SD/-Trp -Leu medium and the interaction between the SS and the WT or mutant LS was scored on the SD/-Leu -Trp-His medium.

### Bacterial Complementation Assay (Screening of Large Subunit Mutants)

The WT or mutant LS cDNA containing pML7 plasmids were sequentially transformed into *E. coli* AC70R1–504 (*glg*C^−^), carrying the SS cDNA expression plasmid pML10. The particular contribution of each mutant to the LS-SS interaction was evaluated by their ability to complement the *glg*C^−^ mutation and synthesize glycogen on Kornberg medium enriched with 2% glucose. Glycogen accumulation phenotypes was detected by iodine staining [52].

### Protein Expression

AC70R1–504 (*glg*C−) cells were grown in 25 ml of LB medium and then induced with 10 mg/L of nalidixic acid and 200 µM isopropyl- b-D-thiogalactopyranoside (IPTG) at room temperature for 20 h when the culture OD_600_ reached 1–1.2. The cells were harvested by centrifugation and disrupted by sonication in 1 ml lysis buffer [1× Tris-buffered saline (TBS), 200 µg/ml lysozyme, 5 mg/ml protease inhibitor (Sigma), and 1 mM phenylmethylsulfonyl fluoride (PMSF) (Roche)]. The crude homogenate was centrifuged at 14,000 g for 10 min. The resulting supernatant was used. Protein levels were determined by Bradford assay (Bradford 1976) according to the manufacturer's (Bio-Rad) instructions (Bio-Rad Laboratories, CA, USA).

### SDS-PAGE and Western Blotting

SDS–PAGE was performed using a Bio-Rad Mini-PROTEAN III electrophoresis cell. Cell lysates containing 10 µg total protein were electrophoresed on a 10% separating gel. Gels were run at 150 V for 1.5 h. After SDS–PAGE, gels were transferred to polyvinylidene difluoride membrane (Biotrace PVDF, Pall Corporation, FL, USA) with a Mini-Trans-Blot electrophoretic transfer cell (Bio-Rad) at 90 V for 1 hr. After pre-blocking with 5% BSA dissolved in Tris-buffered saline (TBS), the membrane was incubated with anti-LS or anti-SS primary antibodies (1∶2000 diluted in 0.15% Tween20/TBS) for 1 hr at room temperature. After a series of washes the membrane was subsequently incubated with HRP-conjugated secondary anti-rabbit IgG antibody (1∶5000 diluted in 0.15% Tween20/TBS) (S41176, Sigma) for 1 hr. Proteins were visualized by Amersham ECL plus western blotting detection system (GE Healthcare, Amersham, UK). The blot was exposed to autoradiography film.

### Native PAGE and Western Blotting

Native-PAGE was performed using a Bio-Rad Mini-PROTEAN III electrophoresis cell. Cell lysates containing 10 µg total protein was mixed with Laemmli's sample loading buffer except β-mercaptoethanol and reducing agent. Samples were electrophoresed on 3–13% polyacrylamide gradient gel (pH 7.0) with 1X running buffer (192 mM Glycine, 25 mM Tris, pH 7.0) at constant 100 V at 4°C for 2 hrs. Western blotting and protein visualization were performed as described above. The observed position of protein complexes was compared with BSA oligomer running pattern.

## References

[1] Ballicora MA, Iglesias AA, Preiss J (2004). ADP-glucose pyrophosphorylase: a regulatory enzyme for plant starch synthesis.. Photosynthesis Research.

[2] Kavakli IH, Slattery CJ, Ito H, Okita TW (2000). The conversion of carbon and nitrogen into starch and storage proteins in developing storage organs: an overview.. Australian Journal of Plant Physiology.

[3] Slattery CJ, Kavakli IH, Okita TW (2000). Engineering starch for increased quantity and quality.. Trends in Plant Science.

[4] Sowokinos JR (1981). Pyrophosphorylases in Solanum-Tuberosum .2. Catalytic Properties and Regulation of Adp-Glucose and Udp-Glucose Pyrophosphorylase Activities in Potatoes.. Plant Physiology.

[5] Sowokinos JR, Preiss J (1982). Pyrophosphorylases in Solanum-Tuberosum .3. Purification, Physical, and Catalytic Properties of Adp-Glucose Pyrophosphorylase in Potatoes.. Plant Physiology.

[6] Okita TW, Nakata PA, Anderson JM, Sowokinos J, Morell M (1990). The Subunit Structure of Potato-Tuber Adpglucose Pyrophosphorylase.. Plant Physiology.

[7] Ballicora MA, Dubay JR, Devillers CH, Preiss J (2005). Resurrecting the ancestral enzymatic role of a modulatory subunit.. Journal of Biological Chemistry.

[8] Nakata PA, Greene TW, Anderson JM, Smithwhite BJ, Okita TW (1991). Comparison of the Primary Sequences of 2 Potato-Tuber Adp-Glucose Pyrophosphorylase Subunits.. Plant Molecular Biology.

[9] Ballicora MA, Laughlin MJ, Fu YB, Okita TW, Barry GF (1995). Adenosine 5′-Diphosphate-Glucose Pyrophosphorylase from Potato-Tuber - Significance of the N-Terminus of the Small-Subunit for Catalytic Properties and Heat-Stability.. Plant Physiology.

[10] Frueauf JB, Ballicora MA, Preiss J (2003). ADP-glucose pyrophosphorylase from potato tuber: site-directed mutagenesis of homologous aspartic acid residues in the small and large subunits.. Plant Journal.

[11] Greene TW, Kavakli IH, Kahn ML, Okita TW (1998). Generation of up-regulated allosteric variants of potato ADP-glucose pyrophosphorylase by reversion genetics.. Proceedings of the National Academy of Sciences of the United States of America.

[12] Kavakli IH, Greene TW, Salamone PR, Choi SB, Okita TW (2001). Investigation of subunit function in ADP-glucose pyrophosphorylase.. Biochemical and Biophysical Research Communications.

[13] Iglesias AA, Barry GF, Meyer C, Bloksberg L, Nakata PA (1993). Expression of the Potato-Tuber Adp-Glucose Pyrophosphorylase in Escherichia-Coli.. Journal of Biological Chemistry.

[14] Kavakli IH, Park JS, Slattery CJ, Salamone PR, Frohlick J (2001). Analysis of allosteric effector binding sites of potato ADP-glucose pyrophosphorylase through reverse genetics.. Journal of Biological Chemistry.

[15] Salamone PR, Greene TW, Kavakli IH, Okita TW (2000). Isolation and characterization of a higher plant ADP-glucose pyrophosphorylase small subunit homotetramer.. Febs Letters.

[16] Hwang SK, Hamada S, Okita TW (2006). ATP binding site in the plant ADP-glucose pyrophosphorylase large subunit.. Febs Letters.

[17] Hwang SK, Hamada S, Okita TW (2007). Catalytic implications of the higher plant ADP-glucose pyrophosphorylase large subunit.. Phytochemistry.

[18] Hwang SK, Nagai Y, Kim D, Okita TW (2008). Direct appraisal of the potato tuber ADP-glucose pyrophosphorylase large subunit in enzyme function by study of a novel mutant form.. Journal of Biological Chemistry.

[19] Cross JM, Clancy M, Shaw JR, Boehlein SK, Greene TW (2005). A polymorphic motif in the small subunit of ADP-glucose pyrophosphorylase modulates interactions between the small and large subunits.. Plant Journal.

[20] Jin XS, Ballicora MA, Preiss J, Geiger JH (2005). Crystal structure of potato tuber ADP-glucose pyrophosphorylase.. Embo Journal.

[21] Giroux MJ, Shaw J, Barry G, Cobb BG, Greene T (1996). A single gene mutation that increases maize seed weight.. Proceedings of the National Academy of Sciences of the United States of America.

[22] Stark DM, Timmerman KP, Barry GF, Preiss J, Kishore GM (1992). Regulation of the Amount of Starch in Plant-Tissues by Adp Glucose Pyrophosphorylase.. Science.

[23] Tuncel A, Kavakli IH, Keskin O (2008). Insights into subunit interactions in the heterotetrameric structure of potato ADP-glucose pyrophosphorylase.. Biophysical Journal.

[24] Liang S, Li L, Hsu WL, Pilcher MN, Uversky V (2009). Exploring the molecular design of protein interaction sites with molecular dynamics simulations and free energy calculations.. Biochemistry.

[25] Jones S, Thornton JM (1997). Analysis of protein-protein interaction sites using surface patches.. J Mol Biol.

[26] Keskin O, Ma B, Nussinov R (2005). Hot regions in protein–protein interactions: the organization and contribution of structurally conserved hot spot residues.. J Mol Biol.

[27] Keskin O, Nussinov R (2007). Similar binding sites and different partners: implications to shared proteins in cellular pathways.. Structure.

[28] Keskin O, Tsai CJ, Wolfson H, Nussinov R (2004). A new, structurally nonredundant, diverse data set of protein-protein interfaces and its implications.. Protein Sci.

[29] Yogurtcu ON, Erdemli SB, Nussinov R, Turkay M, Keskin O (2008). Restricted mobility of conserved residues in protein-protein interfaces in molecular simulations.. Biophys J.

[30] Greene TW, Chantler SE, Kahn ML, Barry GF, Preiss J (1996). Mutagenesis of the potato ADPglucose pyrophosphorylase and characterization of an allosteric mutant defective in 3-phosphoglycerate activation.. Proceedings of the National Academy of Sciences of the United States of America.

[31] Cupp-Vickery JR, Igarashi RY, Perez M, Poland M, Meyer CR (2008). Structural analysis of ADP-glucose pyrophosphorylase from the bacterium Agrobacterium tumefaciens.. Biochemistry.

[32] Bashford D, Case DA (2000). Generalized born models of macromolecular solvation effects.. Annu Rev Phys Chem.

[33] Levy Y, Onuchic JN (2006). Water mediation in protein folding and molecular recognition.. Annu Rev Biophys Biomol Struct.

[34] Greene TW, Hannah LC (1998). Enhanced stability of maize endosperm ADP-glucose pyrophosphorylase is gained through mutants that alter subunit interactions.. Proceedings of the National Academy of Sciences of the United States of America.

[35] Cross JM, Clancy M, Shaw JR, Greene TW, Schmidt RR (2004). Both subunits of ADP-glucose pyrophosphorylase are regulatory.. Plant Physiology.

[36] Iglesias AA, Ballicora MA, Sesma JI, Preiss J (2006). Domain swapping between a cyanobacterial and a plant subunit ADP-glucose pyrophosphorylase.. Plant and Cell Physiology.

[37] Chenna R, Sugawara H, Koike T, Lopez R, Gibson TJ (2003). Multiple sequence alignment with the Clustal series of programs.. Nucleic Acids Res.

[38] Phillips JC, Braun R, Wang W, Gumbart J, Tajkhorshid E (2005). Scalable molecular dynamics with NAMD.. Journal of Computational Chemistry.

[39] Brooks CLI, Karplus M, Pettitt BM, Prigogine I, Rice SA (1988). A theoretical perspective of dynamics structure and thermodynamics..

[40] Jorgensen WL, Chandrasekhar J, Madura JD, Impey RW, Klein ML (1983). Comparison of Simple Potential Functions for Simulating Liquid Water.. Journal of Chemical Physics.

[41] Darden T, York D, Pedersen L (1993). Particle Mesh Ewald - an N.Log(N) Method for Ewald Sums in Large Systems.. Journal of Chemical Physics.

[42] Ryckaert JP, Berendsen HJC (1977). Numerical- ıntegration ofCartesian equations of motion af a System with Constrain Molecular Dynamics of n-alkens.. Journal of Computational Physics.

[43] Feller SE, Zhang YH, Pastor RW, Brooks BR (1995). Constant-Pressure Molecular-Dynamics Simulation - the Langevin Piston Method.. Journal of Chemical Physics.

[44] Hubbard SJ, Thornton JM (1993). ‘NACCESS’, Computer Program.

[45] Kollman PA, Massova I, Reyes C, Kuhn B, Huo SH (2000). Calculating structures and free energies of complex molecules: Combining molecular mechanics and continuum models.. Accounts of Chemical Research.

[46] Srinivasan J, Cheatham TE, Cieplak P, Kollman PA, Case DA (1998). Continuum solvent studies of the stability of DNA, RNA, and phosphoramidate - DNA helices.. Journal of the American Chemical Society.

[47] Onufriev A, Bashford D, Case DA (2000). Modification of the generalized Born model suitable for macromolecules.. Journal of Physical Chemistry B.

[48] Sitkoff D, Sharp KA, Honig B (1994). Accurate Calculation of Hydration Free-Energies Using Macroscopic Solvent Models.. Journal of Physical Chemistry.

[49] Massova I, Kollman PA (2000). Combined molecular mechanical and continuum solvent approach (MM-PBSA/GBSA) to predict ligand binding.. Perspectives in Drug Discovery and Design.

[50] Wang W, Kollman PA (2001). Computational study of protein specificity: The molecular basis of HIV-1 protease drug resistance.. Proceedings of the National Academy of Sciences of the United States of America.

[51] Case DA, Cheatham TE, Darden T, Gohlke H, Luo R (2005). The Amber biomolecular simulation programs.. Journal of Computational Chemistry.

[52] Kavakli IH, Kato C, Choi SB, Kim KH, Salamone PR (2002). Generation, characterization, and heterologous expression of wild-type and up-regulated forms of Arabidopsis thaliana leaf ADP-glucose pyrophosphorylase.. Planta.

